# Direct oral anticoagulants in cancer-associated venous thromboembolism: trial robustness and clinical trade-offs

**DOI:** 10.1016/j.rpth.2026.106844

**Published:** 2026-07-14

**Authors:** Marco Zuin, Cecilia Becattini, Maria Cristina Vedovati, Jean M. Connors, Gregory Piazza

**Affiliations:** 1 Department of Translational Medicine, University of Ferrara, Ferrara, Italy; 2 Division of Cardiology, Padova South Hospitals, Monselice, Padua, Italy; 3 Department of Cardio-Thoraco-Vascular Sciences and Public Health, University of Padova, Padua, Italy; 4 Internal and Cardiovascular Medicine-Stroke Unit, University of Perugia, Perugia, Italy; 5 Hematology Division, Brigham and Women’s Hospital, Harvard Medical School, Boston, Massachusetts, USA; 6 Division of Cardiovascular Medicine, Brigham and Women’s Hospital, Harvard Medical School, Boston, Massachusetts, USA; 7 Thrombosis Research Group, Brigham and Women’s Hospital, Harvard Medical School, Boston, Massachusetts, USA

**Keywords:** cancer, direct oral anticoagulant, prevention, therapy, venous thromboembolism

## Abstract

**Background:**

Although direct oral anticoagulants (DOACs) are often favored over low-molecular-weight heparin for cancer-associated thrombosis, the robustness of trial evidence supporting their use remains uncertain.

**Objectives:**

We evaluated the efficacy, safety, and statistical robustness of DOACs vs dalteparin in patients with cancer-associated venous thromboembolism (VTE).

**Methods:**

We identified phase 3 to 4 randomized controlled trials comparing DOACs with dalteparin in PubMed, SCOPUS, and EMBASE up to August 2025. Primary efficacy was total recurrent VTE; secondary outcomes included recurrent deep vein thrombosis, recurrent PE, and all-cause mortality. Fragility index (FI), reverse FI, and fragility quotients were calculated alongside absolute and relative risk reductions, number needed to treat (NNT), and number needed to harm (NNH).

**Results:**

Five randomized controlled trials including 3055 patients were analyzed. DOACs reduced recurrent VTE across trials (NNT, 17.7-41.9), but benefits were frequently counterbalanced by risk of major bleeding (NNH, 17.6-45). Fragility analyses revealed limited robustness: FI/reverse FI of ≤5 in most efficacy and mortality outcomes. Smaller trials (apixaban and dalteparin in active malignancy associated venous thromboembolism and Selected Cancer Patients at Risk of Recurrence of Venous Thromboembolism) demonstrated higher absolute efficacy (NNT, <20) but extreme fragility (FI, 0-2), whereas larger trials (Hokusai VTE Cancer, Caravaggio, and Cancer Associated Thrombosis, a Pilot Treatment Study Using Rivaroxaban) showed modest absolute benefits with slightly higher stability. Deep vein thrombosis and pulmonary embolism recurrence outcomes were particularly fragile, while mortality effects were inconsistent across studies.

**Conclusions:**

DOACs are viable alternatives to low-molecular-weight heparin for cancer-associated thrombosis, but their comparative efficacy is statistically fragile and offset by bleeding risk. Considering these findings, treatment decisions require an individualized approach, weighing comorbidities and bleeding risk.

## Essentials

• People with cancer have a high risk of venous thromboembolism (VTE).
• Five trials compared oral blood thinners with dalteparin for cancer-related VTE.
• Oral drugs lowered repeat VTE, but many results changed with only a few events.
• Benefits were counterbalanced by bleeding, supporting the need for individualized decision making.

## Graphical abstract

Direct Oral Anticoagulants in Cancer-Associated Venous Thromboembolism: Trial Robustness and Clinical Trade-offs. CRNMB, Clinically relevant non major bleeding; DOAC, Direct oral anticoagulant; DVT, Deep vein thrombosis; FI, Fragility index; PE, Pulmonary embolism; RR, Relative risk; RCT, Randomized controlled trial; RFI, Reverse fragility index; RFQ, Reverse fragility quotient.

**Figure:**
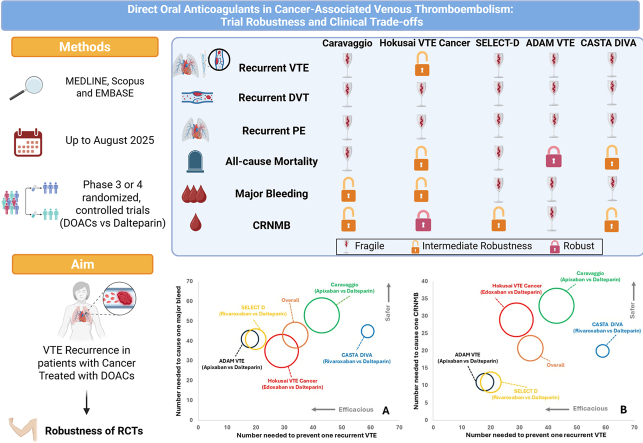

## 1 Introduction

Cancer is one of the strongest risk factors for venous thromboembolism (VTE), accounting for ∼20% of all incident events, with cumulative incidence rates approaching 15% to 20% in high-risk or advanced cancer populations over the disease course [[1], [2], [3], [4], [5], [6], [7]]. The dual risk of thrombosis and bleeding presents unique therapeutic challenges, as patients with cancer are often older, frailer, and burdened by comorbidities, polypharmacy, and organ dysfunction [[8], [9], [10]].

For nearly 2 decades, low-molecular-weight heparin (LMWH) was the standard of care for cancer-associated thrombosis following the Comparison of Low-Molecular-Weight Heparin versus Oral Anticoagulant Therapy for the Prevention of Recurrent Venous Thromboembolism in Patients with Cancer (CLOT) trial, which demonstrated its superiority for prevention of recurrent events over vitamin K antagonists [11]. However, LMWH is limited by the need for daily subcutaneous administration, leading to suboptimal adherence during long-term treatment [8]. The introduction of direct oral anticoagulants (DOACs) offered an attractive alternative with oral dosing, predictable pharmacokinetics, and no routine laboratory monitoring [12].

Since 2018, several randomized controlled trials (RCTs) have compared DOACs with dalteparin in patients with active cancer and acute VTE [[13], [14], [15], [16]]. Collectively, these trials established DOACs as noninferior to LMWH for prevention of recurrent VTE, although concerns regarding increased bleeding, particularly in patients with gastrointestinal or genitourinary malignancies, have persisted [14,17]. Furthermore, the robustness of these outcomes has not been systematically evaluated. Small sample sizes and limited event counts can affect the stability of trial results. In this context, fragility index (FI) and fragility quotient (FQ) analyses provide additional insights into the statistical reliability of RCT outcomes, highlighting how few event changes can alter statistical significance [[18], [19], [20]]. Applying these methods to cancer-associated thrombosis trials may refine the interpretation of efficacy and safety signals and better contextualize the evidence underpinning current guideline recommendations and daily clinical practice.

Therefore, in this study, we assessed the robustness of efficacy and safety outcomes from the 4 pivotal DOAC vs dalteparin RCTs in cancer-associated VTE, to determine the fragility of the findings and clinical meaningfulness of treatment effects for guiding patient care and shared decision making.

## 2 Methods

### 2.1 Data sources and trial selection

We searched PubMed, SCOPUS, and Embase electronic databases from the inception up to August 1, 2025, for phases 3 and 4 RCTs testing DOACs for the prevention of VTEs in patients with cancer. The complete search strategy is detailed in Supplementary Table 1. Eligibility criteria included the following: (i) phase 3 or 4 RCTs; (ii) evaluation of the effect of DOACs in patients with cancer vs dalteparin for the prevention of VTE; and (iii) reporting dichotomous outcomes. To ensure methodological consistency and preserve the validity of fragility analyses, only trials using dalteparin as the comparator were included, as differences in low-molecular-weight heparin type and dosing strategies (eg, enoxaparin) could introduce heterogeneity in anticoagulation intensity and affect the stability of event-based robustness metrics. The Cancer Related VTE Anticoagulation Strategies (CANVAS) trial [21] was excluded from our analysis because ∼82% of patients enrolled received enoxaparin, whereas all other included trials compared DOACs with dalteparin. Therefore, including CANVAS [21] could introduce bias, as enoxaparin requires dose adjustments to achieve target anti-Xa levels, leading to variability in anticoagulation that may influence both efficacy and safety outcomes. Dalteparin, in contrast, provides a more predictable anti-Xa profile with standard weight-based dosing and less frequent dose modifications, resulting in more stable anticoagulation. Only publicly available data were used in this analysis. Accordingly, no institutional review board was needed to approve the study, and informed consent was not required.

### 2.2 Data extraction and quality assessment

Two reviewers (M.Z. and C.B.) independently extracted data concerning study characteristics and event rates from full text journal articles and independently assessed study quality using the Cochrane Risk of Bias Tool (https://methods.cochrane.org/bias/resources/rob-2-revised-cochrane-risk-biastool-randomized-trials).

### 2.3 Outcomes

The primary efficacy end point was recurrent VTE, defined as objectively confirmed deep vein thrombosis (DVT) or pulmonary embolism (PE). Secondary end points included recurrent DVT, recurrent PE, all-cause mortality, major bleeding, and clinically relevant nonmajor bleeding (CRNMB) events. These outcomes were selected for consistency across reviewed RCTs.

### 2.4 Statistical analyses

Continuous variables are presented as means with standard deviations or as median and interquartile range while categorical variables as proportions. For each outcome, we extracted event counts and hazard ratios (HRs) with 95% CIs, when reported from the original publications. FI was calculated for statistically significant results following the method described by Walsh et al. [22]. The FI represents the minimum number of participants whose status would need to be changed (from nonevent to event, or vice versa) in 1 study arm to turn a statistically significant result into a nonsignificant one (*P* < .05 by Fisher exact test). For nonsignificant outcomes, the reverse FI (RFI) was computed by adjusting the number of events in the group with fewer events, while adding nonevents to maintain the total sample size, until the 2-sided Fisher exact test *P* value became <.05 [22]. This approach aligns with prior studies evaluating the robustness of RCT findings using FI and RFI [[23], [24], [25]]. An FI or RFI of ≤5 indicates fragile results, values between 6 and 15 intermediate robustness, and values >15 were considered robust [[21], [22], [23], [24], [25]]. To account for varying trial sizes, FQ and reverse fragility quotient (RFQ) were derived by dividing FI or RFI by the total sample size, providing a proportionate measure of robustness [[21], [22], [23], [24], [25]]. Absolute risk reduction (ARR), number need to treat (NNT) were calculated from event rates, and relative risk (RR) reduction (RRR) was computed as ARR divided by the control group risk. The number needed to harm (NNH) for major bleeding events was also evaluated. The fragility of the meta-analysis results was evaluated according to Atal et al. [25]. All statistical analyses were performed using R version 3.5.1 (R Foundation for Statistical Computing) and SPSS version 20.0 (IBM Corp). Supplementary Table 2 provides formulas used for NNT, ARR, and RRR calculations.

## 3 Results

### 3.1 Patient population

The literature search retrieved a total of 206 articles, after removal of duplicates. By screening titles and abstracts, we identified 184 citations not relevant to our study aims. After a full review of the remaining articles, 5 noninferiority RCTs that included 3055 patients with cancer with acute VTEs, were included in the systematic review and meta-analysis [[14], [15], [16], [17], [18]]. The 5 RCTs included studies were designed to compare the efficacy of DOACs with dalteparin for the treatment of cancer-related acute VTEs [[14], [15], [16], [17], [18]]. Main trial characteristics are outlined in Table 1.

**Table 1:** General characteristics of the population enrolled in randomized controlled trial evaluating the role of direct oral anticoagulants in patients with cancer and VTE.

Characteristic	Caravaggio		Hokusai VTE cancer		SELECT-D		ADAM VTE		CASTA DIVA	
	Apixaban (*n* = 576)	Dalteparin (*n* = 579)	Edoxaban (*n* = 522)	Dalteparin (*n* = 524)	Rivaroxaban (*n* = 203)	Dalteparin (*n* = 203)	Apixaban (*n* = 150)	Dalteparin (*n* = 150)	Rivaroxaban (*n* = 74)	Dalteparin (*n* = 84)
Age (y)	67.2 ± 11.3	67.2 ± 10.9	64.3 ± 11.0	63.7 ± 11.7	67 (22-87)	67 (34-87)	64.4 ± 11.3	64.0 ± 10.8	68.6 (62.9-77.8)	70.7 (62.7-78.7)
Males	292 (50.7)	276 (47.7)	277 (53.1)	263 (50.2)	116 (57.1)	98 (48.2)	72 (48.0)	73 (48.6)	37 (50.0)	40 (47.6)
PE^a^	304 (52.8)	344 (57.7)	328 (62.8)	329 (62.8)	40 (19.7)	38 (18.7)	81 (55.1)	75 (50.7)	67 (90.5)	69 (82.1)
DVT (only)	272 (47.2)	245 (42.3)	194 (37.2)	195 (37.2)	53 (26.1)	57 (28.0)	54 (36.7)	52 (53.1)	6 (8.1)	15 (17.9)
Incidental VTE	116 (20.1)	114 (19.7)	167 (32.0)	173 (33.0)	108^b^ (53.2)	105^b^ (51.7)	NR	NR	36 (48.6)	32 (38.1)
Active cancer	599 (97.0)	565 (97.6)	513 (98.3)	511 (97.5)	NR	NR	NR	NR	74 (100)	84 (100)
Locally advanced or metastatic cancer	389 (67.5)	396 (68.4)	274 (52.5)^b^	280 (53.4)^c^	199 (98.0)	197 (97.0)	96 (65.3)^c^	67 (66.0)^c^	67 (90.5)	70 (83.3)
ECOG score										
0	186 (32.3)	170 (29.4)	97 (17.6)	92 (17.6)	58 (28.5)	61 (30.0)	60 (40.0)	62 (41.3)	NR	NR
1	281 (48.8)	277 (47.8)	148 (28.4)	151 (28.8)	90 (44.3)	95 (46.7)	70 (46.7)	76 (50.7)		
2	109 (18.9)	132 (22.8)	174 (33.3)	159 (30.3)	52 (25.6)	43 (21.1)	20 (13.3)	12 (8.0)		
≥3	—	—	108 (20.7)	122 (23.3)	—	—	—	—		

DVT, deep vein thrombosis; ECOG, Eastern Cooperative Oncology Group; NR, not reported; PE, pulmonary embolism; VTE, venous thromboembolism.

a With or without DVT.

b Defined as incidental PE.

c Defined as metastatic cancer.

The mean age of participants was comparable across studies, ranging from 63.7 to 67.2 years. Patients in Caravaggio, Cancer Associated Thrombosis, a Pilot Treatment Study Using Rivaroxaban (CASTA-DIVA), and Selected Cancer Patients at Risk of Recurrence of Venous Thromboembolism (SELECT-D) were slightly older than those in Hokusai VTE Cancer and apixaban and dalteparin in active malignancy associated venous thromboembolism (ADAM VTE). The proportion of male patients was balanced between treatment arms within each study, varying from ∼48% in ADAM VTE to 57% in SELECT-D. The index VTE presentation differed substantially between trials. In CASTA DIVA, Caravaggio and Hokusai VTE Cancer, more than half of patients presented with PE (52.8%-62.8%), while in SELECT-D, PE was less frequent (19.7%-18.7%). Conversely, in ADAM VTE, PE was reported in about half of participants (55.1% vs 50.7%). Patients with isolated DVT accounted for 37% to 47% in Caravaggio and Hokusai VTE Cancer, 26% to 28% in SELECT-D, and up to 53.1% in the dalteparin arm of ADAM VTE. Of note, the proportion of incidental VTE was highest in SELECT-D (≈53%) and CASTA DIVA (≈40%) intermediate in Hokusai VTE Cancer (32%-33%), and lower in Caravaggio (≈20%); related data were not reported for ADAM VTE.

Almost all patients across studies had active cancer at baseline (97%-98%). The proportion with locally advanced or metastatic disease varied across trials, accounting for approximately two-thirds of patients in Caravaggio (67.5% and 68.4% in the apixaban and dalteparin arms, respectively) and ADAM VTE (65%-66%), just over half in Hokusai VTE Cancer (52.5%-53.4%), the majority in CASTA DIVA (83.3%-90.5%), and nearly all patients in SELECT-D (97%-98%).

Functional status, assessed using the Eastern Cooperative Oncology Group (ECOG) performance score, showed substantial heterogeneity across trials. In Caravaggio, nearly half of the patients had ECOG score of 1 and approximately one-third had ECOG score of 0, whereas only 19% were classified as ECOG score of 2. In contrast, patients enrolled in Hokusai VTE Cancer were more functionally impaired, with approximately one-third having ECOG of 2 and approximately one-fifth with ECOG score of ≥3. In SELECT-D, the majority of patients had good functional status (ECOG 0-1, ∼73%), while 26% had ECOG score of 2. Similarly, in ADAM VTE, >85% of patients were ECOG score 0 to 1, with fewer than 15% classified as ECOG score of 2. The ECOG performance score was not reported in the CASTA DIVA trial.

### 3.2 Recurrent VTE

Across the 5 noninferiority RCTs, the robustness of recurrent VTE outcomes varied substantially. In Caravaggio, the reduction in recurrent VTE with apixaban (5.6% vs 7.9%) was supported by an RFI of 4 and an RFQ of 0.003, indicating a relatively moderate robustness. In Hokusai VTE Cancer, edoxaban vs dalteparin showed a similar pattern (7.9% vs 11.3%), with an RFI of 5 and an RFQ of 0.004. By contrast, the SELECT-D trial demonstrated a more fragile benefit for rivaroxaban (3.9% vs 8.8%), with an FI of only 1 and an RFQ of 0.002. The ADAM VTE trial yielded the largest effect size (0.7% vs 6.3%), yet its robustness was limited to an FI of 2 and an RFQ of 0.006, again highlighting the impact of small sample size. In the CASTA DIVA trial, the reduction of recurrent VTE with rivaroxaban (6.4% vs 10.1%) was associated with an RFI of 1 and an RFQ of 0.006, indicating fragile results (Supplementary Tables 3–7).

### 3.3 Recurrent DVT

For recurrent DVT, FIs were uniformly low. In Caravaggio, the apixaban-to-dalteparin comparison (2.3% vs 2.6%) had an RFI of 1 and an RFQ of 0.0008, underscoring the instability of this finding. Hokusai VTE Cancer showed a statistically significant reduction with edoxaban (3.6% vs 6.7%), but this result was sustained by only a single event change (FI, 1; RFQ, 0.0009). In SELECT-D, rivaroxaban reduced recurrent DVT (1.4% vs 3.4%), yet again with an FI of 1 and an RFQ of 0.002. In ADAM VTE, no events occurred in the apixaban arm, compared with 2.8% on dalteparin, but the FI was 0. Similarly, on the CASTA DIVA trial, no events occurred on the rivaroxaban arm, compared with 3.5% on dalteparin; in both studies, the FI was 0, reflecting extreme sensitivity to event reclassification (Supplementary Tables 3–7).

### 3.4 Recurrent PE

**Figure 1:**
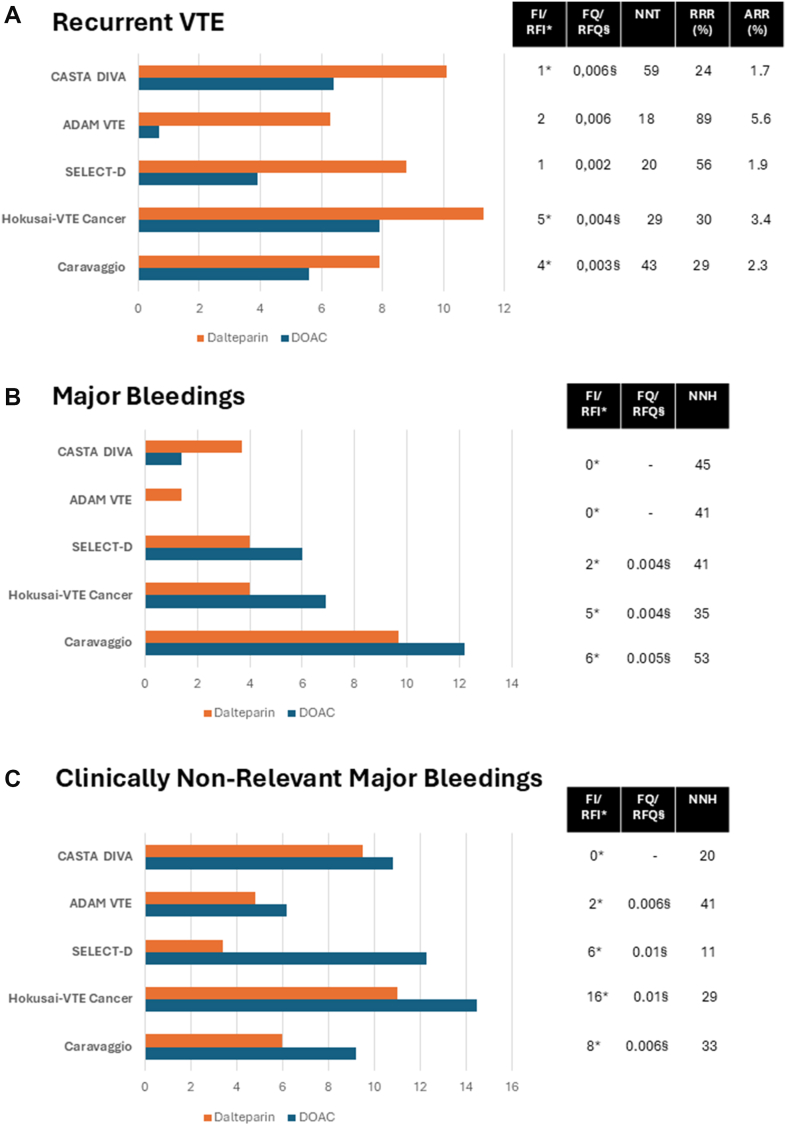
Main and secondary outcomes across the revised randomized controlled trials. (A) Rates of recurrent venous thromboembolism (VTE) events and relative hazard risk in the randomized controlled trials considered for the analysis. (B) Rates of major bleeding events and relative hazard risk in the randomized controlled trials considered for the analysis. (C) Rates of clinically relevant nonmajor bleeding events and relative hazard risk in the randomized controlled trials considered for the analysis. CI, confidence interval; CRNMB, clinically relevant nonmajor bleedings. FI, fragility index; FQ, fragility quotient; NNH, number needed to harm; NNT, number needed to treat; RFI, reverse fragility index; RFQ, reverse fragility quotient; RRR, relative risk reduction. ∗Calculated as RFI. §Calculated as RFQ. For the CASTA DIVA trial, the occurrence of clinically relevant nonmajor bleedings has been calculated as the difference between the composite of major bleedings plus CRNMB − CRNMB.

The robustness of findings for prevention of recurrent PE outcomes also remained limited. In Caravaggio, apixaban reduced PE recurrence (3.3% vs 5.5%), with an RFI of 4 and RFQ of 0.003. In Hokusai VTE Cancer, the comparison between edoxaban and dalteparin (5.2% vs 5.3%) was tenuous, with an RFI of 3 and an RFQ of 0.002. Both SELECT-D and ADAM VTE reported lower PE recurrence with rivaroxaban and apixaban, respectively, but these findings were highly fragile (FI, 1 in SELECT-D; FI, 0 in ADAM VTE). Finally, in the CASTA DIVA, rivaroxaban reduced PE recurrence in 1.3% compared with 2.3% on dalteparin, with an FI of 0 (Supplementary Tables 3–7 and Figure 1A).

### 3.5 All-cause mortality

All-cause mortality outcomes were consistently fragile. In Caravaggio, the apparent reduction with apixaban (23.4% vs 26.4%) was supported by an RFI of 3 and an RFQ of 0.002. In Hokusai VTE Cancer, mortality was numerically higher, but not statistically significant, in the edoxaban arm (39.5% vs 36.6%), with an RFI of 8 and an RFQ of 0.007. Similarly, in CASTA DIVA, the nonsignificant reduction in all-cause mortality (25.7% vs 23.8%) noted with rivaroxaban treatment, was associated an FI of 5 and an RFQ of 0.03. In SELECT-D, the rivaroxaban-vs-dalteparin comparison (23.6% vs 27.5%) was supported by an FI of 1 and an RFQ of 0.002. In ADAM VTE, mortality was numerically higher with apixaban (15.8% vs 10.5%), but the FI was 23, suggesting greater robustness than in the other trials (Supplementary Tables 3–7).

### 3.6 Major bleeding events

In the Caravaggio trial, major bleeding occurred in 22 patients (3.8%) assigned to apixaban and 23 patients (3.9%) assigned to dalteparin (HR, 0.82; 95% CI, 0.40-1.69). The reverse FI was 6 (RFQ, 0.005), corresponding to an NNH of 653, indicating no clinically meaningful excess risk with apixaban. In Hokusai VTE Cancer, major bleeding was more frequent with edoxaban than with dalteparin (36 patients [6.8%] vs 21 patients [4.0%]; HR, 1.77; 95% CI, 1.03-3.04). The RFQ was 0.004, with an NNH of 35, reflecting a clinically significant excess risk. In SELECT-D, major bleeding occurred in 11 patients (5.4%) receiving rivaroxaban and 6 patients (2.9%) receiving dalteparin (HR, 1.83; 95% CI, 0.68-4.96). Although the point estimate favored dalteparin, the CI included unity, and the RFI was low (2), with an RFQ of 0.004, suggesting a fragile result. In ADAM VTE, no major bleeding events occurred in the apixaban group, compared with 2 events (1.3%) in the dalteparin group (HR, 0.93; 95% CI, 0.43-2.02). The RFI was 0, and the NNH was 41, supporting the safety of apixaban in this population. Similarly, in CASTA DIVA, only 1 bleeding event was registered in the rivaroxaban arm (1.4% vs 3.7%) compared with dalteparin (HR, 0.36; 95% CI, 0.04-3.43), with an RFI of 0 and an NNT of 45 (Figure 1B).

### 3.7 Clinically relevant nonmajor bleeding

CRNMB rates were numerically higher with DOACs in most trials. In Caravaggio, CRNMB occurred in 53 patients (9.2%) in the apixaban group vs 35 patients (6.0%) in the dalteparin group (HR, 1.42; 95% CI, 0.88-2.30; RFQ, 0.006). Similarly, Hokusai VTE Cancer showed an increased risk of CRNMB with edoxaban (76 patients [14.5%] vs 58 [11.0%]; HR, 1.40; 95% CI, 1.03-1.89), with a robust RFQ of 0.01 and an NNH of 29. In SELECT-D, CRNMB was substantially higher with rivaroxaban than with dalteparin (25 patients [12.3%] vs 7 [3.4%]; HR, 3.76; 95% CI, 1.63-8.69), with an RFI of 6 and an RFQ of 0.01, indicating a clinically important signal for excess bleeding risk. Similar findings were observed in CASTA DIVA trial, with a higher but not statistically significant occurrence of CRNMB in the rivaroxaban arm compared with that in dalteparin arm (9 patients [12.2%] vs 8 [9.8%]; HR, 1.27; 95% CI, 0.49-3.26). Finally, in ADAM VTE, CRNMB occurred in 9 patients (6.2%) in the apixaban group vs 7 patients (4.8%) in the dalteparin group (RFI, 2; RFQ, 0.006), without a significant difference between groups (Supplementary Tables 3–7 and Figure 1C).

### 3.8 Pooled analysis

The pooled analysis demonstrated a statistically significant reduction in recurrent VTE among patients with cancer treated with apixaban compared with dalteparin (5.6% vs 8.9%; RR, 0.65, 95% CI, 0.50-0.84; *I*^2^ = 1%). This corresponded to a RRR of 37%, an ARR of 3.3%, and an NNT of 30. The robustness of this finding was supported by an FI of 21 and an FQ of 0.006.

When stratified by VTE subtype, DOACs significantly lowered the risk of recurrent DVT (2.3% vs 4.1%; RR, 0.58; 95% CI, 0.39-0.88; *I*^2^ = 0%), with an RRR of 45%, ARR of 1.9%, and NNT of 53 (FI, 6; FQ, 0.002). A nonsignificant trend toward reduction was observed for recurrent PE (3.3% vs 4.6%; RR, 0.72; 95% CI, 0.51-1.03; *I*^2^ = 0%), with an NNT of 77, ARR of 1.3%, and RRR of 28%.

**Figure 2:**
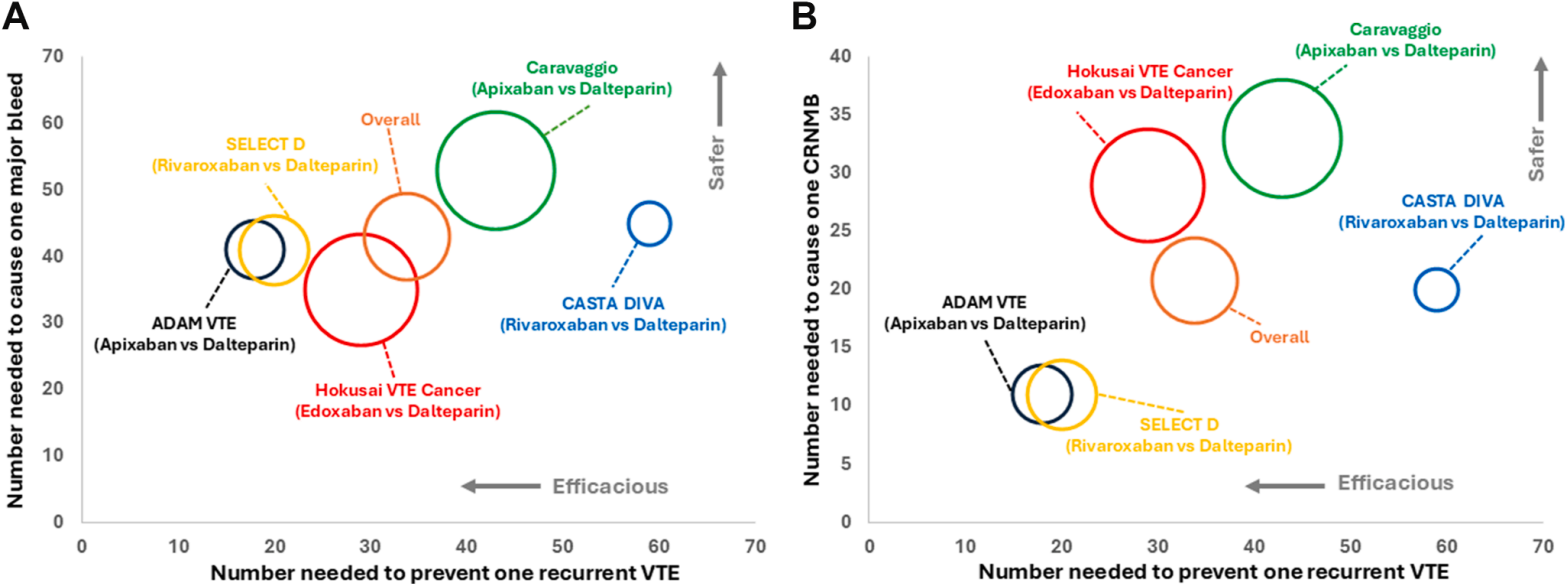
Trial-specific and pooled outcomes for direct oral anticoagulants (DOACs) vs dalteparin in patients with cancer-associated venous thromboembolism (VTE). Panels show event rates, number needed to harm (NNT), and number needed to treat (NNH) for recurrent VTE, major bleeding (A), and CRNMB (B) across individual randomized controlled trials and the pooled analysis. Differences in the position of individual trials reflect variation in effect size, event rates, study precision, and fragility metrics, driven by heterogeneity in patient populations and sample size rather than differences in the treatment comparison itself. Fragility indices (fragility index/reverse fragility index) and fragility quotients (fragility quotient/reverse fragility quotient) are indicated for each trial where applicable. Note that *y*-axis scales differ between panels to reflect absolute event rates; direct visual comparison across outcomes should be avoided. Pooled estimates are presented alongside trial-specific data to contextualize both the overall treatment effect and its statistical robustness.

All-cause mortality did not differ significantly between groups (28.3% vs 28.4%; RR, 1.00; 95% CI, 0.87-1.15; *I*^2^ = 21%; RFI, 0) with fragile results. Major bleeding events occurred in 70 patients (4.6%) treated with DOAC and in 55 patients (3.5%) receiving dalteparin (RR, 1.26; 95% CI, 0.80-2.00; *I*^2^ = 24%). The RFI was 13 (RFQ, 0.004), yielding an NNH of 97. CNRMB was significantly more frequent with DOAC therapy (172 patients [11.3%] vs 115 [7.4%]; RR, 1.53; 95% CI, 1.14-2.03; *I*^2^ = 24%). This result was robust (RFI, 26; RFQ, 0.82), with an NNH of 26, indicating a consistent excess risk of CRNMB among patients across studies (Figure 2, Table 2, and Supplementary Figures 1 and 2).

**Table 2:** Robustness of outcomes from the pooled analysis of the considered randomized trials.

Outcome	Pooled analysis								
	DOAC (*n* = 1520)	Dalteparin (*n* = 1535)	RR (95% CI)	*I*^2^ (%)	FI/FRI^a^	FQ/RFQ^b^	NNT or NNH^c^	RRR (%)	ARR (%)
Recurrent VTE	86 (5.6)	138 (8.9)	0.65 (0.50-0.84)	1	21	0.006	30	37	3.3
Recurrent DVT	35 (2.3)	64 (4.1)	0.58 (0.39-0.88)	0	9	0.002	53	45	1.9
Recurrent PE	51 (3.3)	72 (4.6)	0.72 (0.51-1.03)	0	21^a^	0.006^b^	77	28	1.3
All-cause mortality	431 (28.3)	436 (28.4)	1.00 (0.87-1.15)	21	0^a^	0^b^	500	1	0.2
Major bleeding	70 (4.6)	55 (3.5)	1.26 (0.80-2.00)	24	13^a^	0.004^b^	97^c^	—	—
CRNMB	172 (11.3)	115 (7.4)	1.53 (1.14-2.03)	24	26	0.008	26^c^	—	—

ARR, absolute risk reduction; CI, confidence interval; CRNMB, clinically relevant nonmajor bleeding; DVT, deep vein thrombosis; FI, fragility index; FQ, fragility quotient; NNH, number needed to harm; NNT, number needed to treat; NR, not reported; PE, pulmonary embolism; RFI, reverse fragility index; RR, relative risk; RRR, relative risk reduction; VTE, venous thromboembolism.

a Calculated as RFI.

b Calculated as RFQ.

c Number needed to harm.

## 4 Discussion

In this evaluation of noninferiority RCTs comparing DOACs with dalteparin for cancer-associated VTE, fragility analysis demonstrated that comparative efficacy signals were statistically fragile, with FI and RFI values of ≤5 in nearly all cases, indicating that a handful of outcome changes could negate significance. Mortality findings were uniformly fragile, except in ADAM VTE, where a higher FI supported the observed survival difference. When integrated with NNT and NNH calculations, these results highlight the narrow therapeutic balance of DOACs: across trials, the NNT to prevent 1 recurrent VTE closely paralleled NNH for a major or fatal bleed, emphasizing a delicate trade-off in efficacy and safety [[14], [15], [16], [17], [18]]. These findings demonstrate that, while DOACs are effective, their benefit is frequently counterbalanced by CRNMB, particularly in smaller trials with high event rates but statistically fragile findings.

In the pooled analysis, treatment with DOACs was associated with a clinically meaningful reduction in recurrent VTE when compared with dalteparin, with an NNT of 37 and an FI of 21, suggesting that the findings were statistically robust. The reduction was primarily driven by a lower incidence of recurrent DVT, with an even more favorable NNT and an extremely low FQ, indicating stability of the result. In contrast, the apparent reduction in recurrent PE did not meet conventional thresholds for statistical significance. The outcome was robust highly (RFI, 21), consistent with prior observations from RCTs in which the benefit of DOACs for PE recurrence was less pronounced and often lacked robustness. All-cause mortality did not differ meaningfully between groups, as previously demonstrated by individual RCTs included into the analysis. Major bleeding rates were similar between DOACs and dalteparin, with an NNH of 97, supporting the overall safety profile of DOACs in this high-risk population. Across trials, CRNMB occurred more frequently with DOACs compared with dalteparin, reaching statistical significance in Hokusai VTE Cancer [17] and SELECT-D [16] and showing a consistent direction of effect in Caravaggio [14], CASTA DIVA [18], and the pooled analysis. In this regard, the Apixaban Cancer–Associated Thrombosis (API-CAT) trial demonstrated that, among patients with active cancer who had completed at least 6 months of anticoagulation for proximal DVT or PE, extended treatment with reduced-dose apixaban (2.5 mg twice daily) was noninferior to full-dose apixaban (5 mg twice daily) for the prevention of recurrent VTE, while being associated with a lower incidence of clinically relevant bleeding [26].

An important and unifying feature of the RCTs evaluating DOACs for cancer-associated VTE is their noninferiority design, with the notable exception of ADAM VTE trial [15], which was primarily conceived as a safety study. This methodological choice is not coincidental but reflects a deliberate shift in the therapeutic priorities of this clinical setting. Given the well-established efficacy of LMWH, the principal unmet need is no longer incremental gains in antithrombotic efficacy but, rather, the optimization of treatment acceptability, persistence, and safety over prolonged treatment durations. Moreover, the emphasis on noninferiority is closely aligned with safety considerations. In a population intrinsically at high risk of bleeding, particularly gastrointestinal and genitourinary hemorrhage, demonstrating comparable efficacy while potentially reducing clinically relevant bleeding may be of greater clinical value than achieving marginal superiority in preventing recurrent VTE. Thus, the preference for noninferiority frameworks underscores a broader conceptual shift from efficacy maximization to harm minimization and patient-centered care.

An important feature of our analysis is the use of FIs alongside traditional effect measures such as NNT, NNH, ARR, and RRR. We acknowledge that fragility metrics are inherently study-specific and assume a uniform risk across participants, which does not fully capture the heterogeneity in baseline risk, functional status, or cancer type among trial populations. Consequently, FI and FQ values should be interpreted primarily as descriptive indicators of statistical robustness within individual trials; however, they can also be extended to meta-analyses and pooled analyses within established methodological frameworks, with appropriate consideration of between-study heterogeneity. This fragility analysis highlights that many observed benefits of DOACs, particularly in smaller trials with low event rates, are sensitive to a small number of outcome changes. This observation is consistent with the rarity of recurrent VTE, DVT, and PE events and the clinical heterogeneity of patients enrolled in these trials. In this context, FIs complement conventional efficacy metrics by quantifying how easily statistically significant findings could change with minimal alterations in event counts. From a clinical perspective, integrating FIs with NNT and NNH provides a more nuanced understanding of the therapeutic trade-offs in cancer-associated thrombosis. Even when DOACs show favorable effect sizes, the limited robustness of some trial outcomes underscores the need for individualized decision making, weighing both recurrence prevention and bleeding risk. While our analysis does not provide new efficacy estimates beyond those reported in prior meta-analyses, it offers a complementary lens on the certainty of existing evidence. These insights can support shared decision making, particularly for patients at higher risk of bleeding or with comorbidities that influence the balance of benefit and harm. Overall, fragility metrics should be interpreted alongside traditional trial outcomes. They do not replace effect size estimates or HRs but provide an intuitive and clinically relevant tool to contextualize the reliability of trial results, especially for rare events in heterogeneous populations.

Taken together, these results indicate that DOACs confers a durable and clinically relevant reduction in recurrent VTE, particularly recurrent DVT, without a significant increase in major bleeding. The effect on PE recurrence remains uncertain, and mortality effects are heterogeneous [27]. These findings are consistent with previous randomized trials of DOACs in cancer-associated thrombosis and support their use as a standard of care while underscoring the need for further study of such agents specifically for PE prevention and improving survival.

Our analysis underscores that trial heterogeneity strongly influences efficacy and fragility outcomes. Caravaggio [14] and Hokusai VTE Cancer [17] studies predominantly enrolled patients with symptomatic PE and a broad spectrum of cancers, providing large-scale, stable but modest effect estimates. The CASTA DIVA trial [18] enrolled patients with cancer or treated within the previous 6 months, which lead to the inclusion of ∼23% of patients whose cancer was cured at the time of enrolment. In contrast, SELECT-D, disproportionately enrolled patients with incidental VTE and nearly universal metastatic disease, amplifying event rates but yielding fragile statistical support [16]. ADAM VTE included a smaller, healthier cohort, reflected in lower mortality and narrower efficacy–safety margins [15]. These baseline differences are critical for interpreting discrepancies in NNT, NNH, and FIs across trials.

From a clinical perspective, the close alignment of NNT and NNH values across trials illustrates that the benefit of preventing 1 recurrent VTE is often counterbalanced by the risk of inducing 1 major or fatal bleed. Smaller studies (ADAM VTE [15] and SELECT-D [16]) suggested greater efficacy (NNT, <20) but were sustained by FI values of 1 to 2, questioning the reliability of their results. Larger studies (Hokusai VTE Cancer [17], Caravaggio [14], and CASTA DIVA [18]) demonstrated attenuated absolute benefits (NNT, ∼30-60) but provided more generalizable estimates. Fragility analysis may therefore caution enthusiasm for DOAC superiority and emphasizes the importance of interpreting efficacy signals considering sample size, event rates, and baseline risk. Indeed, in our results, FI and FQ can diverge from ARR and NNT, highlighting instances in which statistical significance is tenuous despite favorable effect sizes [28]. Understanding these nuances can also help clinicians communicate risks and benefits more transparently, supporting shared decision making with patients based on both absolute and relative outcomes.

A potential extension of the present work would be the application of fragility analysis to earlier randomized trials comparing LMWH with VKA in cancer-associated thrombosis. Although these studies established the historical standard of care, their relatively modest sample sizes and event rates suggest that an evaluation of their statistical robustness using fragility metrics could provide additional insight into the strength of the original evidence base. However, such an analysis falls beyond the scope of the present study, which was specifically designed to assess the robustness of contemporary trials informing current clinical practice.

Our findings should be interpreted in the context of current guideline recommendations. Both the NCCN Clinical Practice Guidelines in Oncology (2025) for Venous Thromboembolic Disease [29] and the American Society of Hematology (2022) guidelines [1] recommend DOACs over LMWH for most patients with cancer-associated thrombosis who do not have high-risk gastrointestinal or gastroesophageal lesions, while emphasizing individualized therapy based on bleeding risk, comorbidities, patient comfort, and individual preferences. The reduction in recurrent VTE observed across trials in our analysis (NNT, 17.7-41.9) aligns with these recommendations, but the concomitant risk of major bleeding (NNH, 17.6-45) underscores the narrow therapeutic window in certain patient populations. Importantly, FIs and NNT/NNH metrics reflect study-specific limitations, including small event counts, patient heterogeneity, and noninferiority trial designs and, therefore, should be interpreted as complementary to standard efficacy and safety measures rather than definitive evidence.

These findings have several implications for practice. First, the consistent reduction in recurrent VTE supports the use of DOACs as an acceptable, and often preferred, alternative to LMWH in many patients with cancer-associated thrombosis, given the practical advantages of oral administration, avoidance of daily injections, and avoidance of routine laboratory monitoring. Although concerns have been raised about potentially lower adherence to LMWH, a recent propensity score–matched analysis demonstrated comparably high medication adherence with both strategies, with proportions of days covered approaching 95% [30]. Importantly, treatment persistence was significantly longer with DOACs, with a median duration exceeding that of LMWH by >80 days, suggesting improved long-term treatment continuity with oral anticoagulation [30]. Second, the narrow alignment of NNT and NNH highlights the limited therapeutic window; clinicians must weigh the benefit of VTE prevention against the patient’s bleeding risk, particularly in those with unresected gastrointestinal or genitourinary malignancies [31]. Third, fragility analyses caution against overinterpreting efficacy advantages from smaller trials. Major international guidelines recommend DOACs as first-line therapy while emphasizing careful patient selection, and our results reinforce this nuanced approach [32,33]. Shared decision making remains central, ensuring that anticoagulation aligns with individual goals, cancer type, and patient preferences. However, no single DOAC emerges as universally optimal; trial-level heterogeneity and statistical fragility highlight the need for patient-specific decision making that considers cancer type, other thrombotic risk factors, bleeding risk, comorbidities, and patient preference. The consistent NNT–NNH alignment indicates that anticoagulant choice should be guided by individual risk–benefit assessment and patient preference rather than presumed superiority.

This study provides additional, clinically relevant insights specifically focused on the robustness and interpretation of DOACs vs dalteparin in cancer-associated thrombosis. First, fragility analysis reveals that the apparent efficacy signals for recurrent VTE in individual trials are frequently dependent on a small number of events, highlighting the limited statistical stability of several study-level findings. Second, when these data are synthesized, the pooled estimates show a more consistent and robust reduction in recurrent VTE, particularly for DVT, supporting a reliable class effect of DOACs in preventing thrombotic recurrence. Third, the parallel assessment of NNT and NNH quantifies the delicate balance between thrombotic protection and bleeding risk. Finally, the observed intertrial variability in patient populations and event patterns explains part of the heterogeneity in both effect sizes and fragility metrics, reinforcing that treatment decisions should be individualized rather than based solely on aggregated averages. Together, these findings refine the interpretation of DOAC efficacy and safety in cancer-associated VTE by explicitly linking statistical robustness to clinically meaningful treatment trade-offs.

### 4.1 Limitations

This study has several limitations. First, FIs do not account for time-to-event dynamics, which are central in VTE trials. Two treatment groups may have identical event rates but different survival curves and yet yield similar fragility metrics. However, prior methodological analyses suggest that results using these indices are generally comparable when applied to time-to-event vs frequency data, indicating that this limitation may not substantially alter interpretations of robustness in most settings [21]. Second, because RCTs are typically powered for primary outcomes, fragility metrics for secondary end points or subgroup analyses should be considered exploratory. Furthermore, small sample sizes or low event rates may limit meaningful interpretation. Moreover, FI and RFI calculations rely on Fisher exact test, which may not fully align with the statistical methods used in the original trials, such as Cox proportional hazards models with covariate adjustment, potentially leading to discrepancies between recalculated and reported *P* values. Additionally, the CANVAS trial was excluded from the main analysis; while its inclusion may have introduced additional heterogeneity due to differences in design and population characteristics, its exclusion limits the generalizability of the findings to studies specifically evaluating dalteparin as the comparator LMWH [21]. FIs, while providing a more intuitive assessment than dichotomous *P* values alone, remain within a frequentist framework and do not incorporate effect size or clinical relevance. They should therefore be interpreted alongside ARR and RRR, NNT, and trial context. Differences in trial design, including protocolized management of thrombocytopenia and shorter treatment exposure in dalteparin arms, potentially related to lower adherence with LMWH compared with DOACs, may have influenced both bleeding and efficacy outcomes and should be considered when interpreting effect estimates and their statistical robustness. Our findings are further limited by heterogeneity in trial design, eligibility criteria, and patient mix, and fragility analysis may disproportionately affect smaller studies. Finally, all included trials used dalteparin as the comparator, limiting generalizability to other LMWHs. Alternative approaches, such as Bayesian inference, could offer a more comprehensive evaluation of treatment effect but are not yet widely applied to VTE trials.

## 5 Conclusion

Direct oral anticoagulants are a safe and effective alternative to dalteparin for cancer-associated thrombosis, consistently reducing recurrent VTE, although these efficacy signals are statistically fragile. When considered alongside aligned NNT and NNH values, these data highlight both the promise and limitations of DOACs in this setting. Anticoagulant choice should remain individualized, balancing recurrence risk, bleeding proclivity, and patient preference.
