# Supplementary material for: Direct oral anticoagulants in cancer-associated venous thromboembolism: trial robustness and clinical trade-offs
Source: Res Pract Thromb Haemost. 2026 Jul 14;10(5):106844. doi: 10.1016/j.rpth.2026.106844 (PMC13487287; doi:10.1016/j.rpth.2026.106844)
Supplement: Supplementary file 1 [file mmc1.docx]

**Direct Oral Anticoagulants in Cancer-Associated Venous Thromboembolism: Trial Robustness and Clinical Trade-offs**

**Running Head:** Fragility of DOACs in Cancer VTE

Marco Zuin^1,2,3^ MD MS, Cecilia Becattini^4^ MD PhD, Maria Cristina Vedovati^4^ MD PhD, Jean M Connors^5^ MD, Gregory Piazza^6,7^ MD PhD

^1^ Department of Translational Medicine, University of Ferrara, Ferrara, Italy.

^2^ Division of Cardiology, South Padova General Hospitals, Monselice, Padua, Italy.

^3^ Department of Cardio-Thoraco-Vascular Sciences and Public Health, University of Padova, Padua, Italy.

^4^ Internal and Cardiovascular Medicine-Stroke Unit, University of Perugia, Perugia, Italy

^5^ Hematology Division, Brigham and Women's Hospital, Harvard Medical School, Boston, Massachusetts, USA

^6^ Division of Cardiovascular Medicine, Brigham and Women's Hospital, Harvard Medical School, Boston, Massachusetts, USA

^7^ Thrombosis Research Group, Brigham and Women's Hospital, Harvard Medical School, Boston, Massachusetts, USA

**Supplementary Files**

[**Supplementary Table 1.** Search Strategy.	2](#_Toc217809194)

[**Supplementary Table 2.** Formulas used for the calculation of different measures in the study.	3](#_Toc217809195)

[**Supplementary Table 3:** Robustness of outcomes from the CARAVAGGIO trial.	4](#_Toc217809196)

[**Supplementary Table 4:** Robustness of outcomes from the Hokusai Cancer VTE trial..	5](#_Toc217809197)

[**Supplementary Table 5:** Robustness of outcomes from the SELECT-D trial..	6](#_Toc217809198)

[**Supplementary Table 6:** Robustness of outcomes from the SELECT-D trial. ARR:.	7](#_Toc217809199)

[**Supplementary Table 7:** Robustness of outcomes from the CASTA DIVA trial..	8](#_Toc217809200)

[**Supplementary Figure 1.** Risk of Bias Assessment of Selected Trials using the Cochrane risk of bias tool for randomized trials (Version 2).	9](#_Toc217809201)

[**Supplementary Figure 2.** Forest plot of outcomes from the included randomized controlled trials.: Venous thromboembolism.	11](#_Toc217809202)

[**Supplementary Figure 3.** Funnel plot for the main outcomes of the considered randomized controlled trials.	12](#_Toc217809203)

# **Supplementary Table 1.** Search Strategy.

| Database | Search string |
| --- | --- |
| PubMed/MEDLINE | (("Cancer"[Mesh] OR cancer* OR neoplasm*OR tumor*) AND ("Venous Thromboembolism"[Mesh] OR "VTE" OR "Deep Vein Thrombosis" OR DVT OR "Pulmonary Embolism" OR PE) AND ("Direct Oral Anticoagulants" OR DOAC* OR "Novel Oral Anticoagulants" OR NOAC* OR apixaban OR rivaroxaban OR edoxaban OR dabigatran) AND ("Low-Molecular-Weight Heparin" OR LMWH) AND ("Randomized Controlled Trial"[Publication Type] OR "Randomized"[Title/Abstract] OR "Randomised"[Title/Abstract] OR "RCT"[Title/Abstract])) |
| SCOPUS | (TITLE-ABS-KEY(cancer OR neoplasm* OR malignan* OR tumor* OR tumour*) AND TITLE-ABS-KEY("venous thromboembolism" OR VTE OR "deep vein thrombosis" OR DVT OR "pulmonary embolism" OR PE) AND TITLE-ABS-KEY("direct oral anticoagulant" OR DOAC* OR "novel oral anticoagulant" OR NOAC* OR apixaban OR rivaroxaban OR edoxaban OR dabigatran) AND TITLE-ABS-KEY("low-molecular-weight heparin" OR LMWH OR) AND (RCT OR "randomized controlled trial" OR "randomised controlled trial")) |
| EMBASE | ('cancer'/exp OR cancer* OR neoplasm* OR malignan* OR tumor* OR tumour*) AND ('venous thromboembolism'/exp OR 'venous thromboembolism' OR VTE OR 'deep vein thrombosis' OR DVT OR 'pulmonary embolism' OR PE) AND ('direct oral anticoagulant'/exp OR 'direct oral anticoagulant' OR DOAC* OR 'novel oral anticoagulant' OR NOAC* OR apixaban OR rivaroxaban OR edoxaban OR dabigatran) AND ('low-molecular-weight heparin'/exp OR 'low-molecular-weight heparin' OR LMWH) AND ('randomized controlled trial'/exp OR 'randomized controlled trial' OR RCT OR randomized) |

# **Supplementary Table 2.** Formulas used for the calculation of different measures in the study.

| **Measure** | **Abbreviation** | **Formula** |
| --- | --- | --- |
| Adverse events rate in the experimental group | AER | - |
| Absolute Risk | AR | (number of events (good or bad) in treated or control group)/ (number of patients in that group) |
| Absolute risk increase | ARI | 1/ (AER-CER) |
| Absolute Risk in the Treatment group | ART | AR of events in the treatment group |
| Absolute Risk in the Control group | ARC | AR of events in the control group |
| Absolute Risk Reduction | ARR | ARC - ART |
| Adverse events in the control group | CER | - |
| Relative Risk | RR | 1 - RRR |
| Relative Risk Reduction | RRR | 1- RR |
| Number Needed to Treat | NTT | 1/ARR |
| Number Needed to Harm | NNH | 1/ARI |

# **Supplementary Table 3:** Robustness of outcomes from the Caravaggio trial. ARR: Absolute Risk reduction; CI: Confidence interval; CRNMB: Clinically relevant non-major bleeding; DVT: Deep vein thrombosis; FI: Fragility index; FQ: Fragility quotient; HR: Hazard ratio; NNH: Number needed to harm; NNT: Number needed to treat; PE: Pulmonary embolism; RFI: Reverse fragility index; RRR: Relative Risk Reduction; VTE: Venous thromboembolism; *Calculated as RFI. §Calculated as RFQ.

|  | **Caravaggio** | | | | | | | |
| --- | --- | --- | --- | --- | --- | --- | --- | --- |
|  | **Apixaban**  **N=576** | **Dalteparin**  **N=579** | **HR** | **FI/**  **RFI*** | **FQ/**  **RFQ§** | **NNT**  **Or NNH°** | **RRR**  **(%)** | **ARR**  **(%)** |
| **Recurrent**  **VTE** | 32  (5.6) | 46  (7.9) | 0.63  [0.37-1.07] | 4* | 0.003§ | 43 | 29 | 2.3 |
| **Recurrent**  **DVT** | 13  (2.3) | 15  (2.6) | 0.87  [0.34-2.21] | 1* | 0.0008§ | 333 | 12 | 0.3 |
| **Recurrent**  **PE** | 19  (3.3) | 32  (5.5) | 0.54  [0.29-1.03] | 4* | 0.003§ | 45 | 40 | 2.2 |
| **All-cause mortality** | 135  (23.4) | 153  (26.4) | 0.82  [0.62-1.09] | 3* | 0.002§ | 33 | 11 | 3 |
| **Major Bleeding** | 22  (3.8) | 23  (3.9) | 0.82  [0.40-1.69] | 6* | 0.005§ | 53° | - | - |
| **CRNMB** | 53  (9.2) | 35  (6.0) | 1.42  [0.88-2.30] | 8* | 0.006§ | 33° | - | - |

# **Supplementary Table 4:** Robustness of outcomes from the Hokusai VTE Cancer trial. ARR: Absolute Risk reduction; CI: Confidence interval; CRNMB: Clinically relevant non-major bleeding; DVT: Deep vein thrombosis; FI: Fragility index; FQ: Fragility quotient; HR: Hazard ratio; NNH: Number needed to harm; NNT: Number needed to treat; PE: Pulmonary embolism; RFI: Reverse fragility index; RRR: Relative Risk Reduction; VTE: Venous thromboembolism; *Calculated as RFI. §Calculated as RFQ.

|  | **HOKUSAI VTE Cancer** | | | | | | | |
| --- | --- | --- | --- | --- | --- | --- | --- | --- |
|  | **Edoxaban**  **N=522** | **Dalteparin**  **N=524** | **HR** | **FI/RFI*** | **FQ/**  **RFQ§** | **NNT**  **or NNH°** | **RRR**  **(%)** | **ARR**  **(%)** |
| **Recurrent**  **VTE** | 41  (7.9) | 59  (11.3) | 0.71  [0.48-1.06] | 5* | 0.004§ | 29 | 30 | 3.4 |
| **Recurrent**  **DVT** | 19  (3.6) | 35  (6.7) | 0.56  [0.32-0.97] | 1 | 0.0009§ | 32 | 46 | 3.1 |
| **Recurrent**  **PE** | 27  (5.2) | 28  (5.3) | 1.00  [0.59-1.69] | 3* | 0.002§ | 1000 | 2 | 0.1 |
| **All-cause mortality** | 206  (39.5) | 192  (36.6) | 1.12  [0.92-1.37] | 8* | 0.007§ | 34 | 8 | 2.9 |
| **Major Bleeding** | 36  (6.8) | 21  (4.0) | 1.77  [1.03-3.04] | 5§ | 0.004§ | 35 | - | - |
| **CRNMB** | 76  (14.5) | 58  (11.0) | 1.40  [1.03-1.89] | 16§ | 0.01§ | 29 | - | - |

# **Supplementary Table 5:** Robustness of outcomes from the SELECT-D trial. ARR: Absolute Risk reduction; CI: Confidence interval; CRNMB: Clinically relevant non-major bleeding; DVT: Deep vein thrombosis; FI: Fragility index; FQ: Fragility quotient; HR: Hazard ratio; Number needed to harm; NNT: Number needed to treat; NR: Not reported; PE: Pulmonary embolism; RFI: Reverse fragility index; RRR: Relative Risk Reduction; VTE: Venous thromboembolism; *Calculated as RFI. §Calculated as RFQ.

|  | **SELECT-D** | | | | | | | |
| --- | --- | --- | --- | --- | --- | --- | --- | --- |
|  | **Rivaroxaban**  **N=203** | **Dalteparin**  **N=203** | **HR** | **FI/**  **FRI*** | **FQ/**  **RFQ§** | **NNT**  **or NNH°** | **RRR (%)** | **ARR (%)** |
| **Recurrent**  **VTE** | 8  (3.9) | 18  (8.8) | 0.43  [0.19-0.99] | 1 | 0.002 | 20 | 56 | 5 |
| **Recurrent**  **DVT** | 3  (1.4) | 7  (3.4) | NR | 1* | 0.002§ | 53 | 56 | 1.9 |
| **Recurrent**  **PE** | 4  (1.9) | 9  (4.4) | NR | 1* | 0.002§ | 42 | 55 | 2.4 |
| **All-cause mortality** | 48  (23.6) | 56  (27.5) | NR | 1* | 0.002§ | 25 | 14 | 4 |
| **Major Bleeding** | 11  (5.4) | 6  (2.9) | 1.83  [0.68-4.96] | 2* | 0.004§ | 41 | - | - |
| **CRNMB** | 25  (12.3) | 7  (3.4) | 3.76  [1.63-8.69] | 6* | 0.01§ | 11 | - | - |

# **Supplementary Table 6:** Robustness of outcomes from the SELECT-D trial. ARR: Absolute Risk reduction; CI: Confidence interval; CRNMB: Clinically relevant non-major bleeding; DVT: Deep vein thrombosis; FI: Fragility index; FQ: Fragility quotient; HR: Hazard ratio; NNH: Number needed to harm; NNT: Number needed to treat; NR: Not reported PE: Pulmonary embolism; RFI: Reverse fragility index; RRR: Relative Risk Reduction; VTE: Venous thromboembolism; *Calculated as RFI. §Calculated as RFQ.

|  | **ADAM VTE** | | | | | | | |
| --- | --- | --- | --- | --- | --- | --- | --- | --- |
|  | **Apixaban**  **N=145** | **Dalteparin**  **N=145** | **HR** | **FI/**  **FRI*** | **FQ/**  **RFQ§** | **NNT**  **or NNH°** | **RRR (%)** | **ARR (%)** |
| **Recurrent**  **VTE** | 1  (0.7) | 9  (6.3) | 0.09  [0.01-0.78] | 2 | 0.006 | 18 | 89 | 5.6 |
| **Recurrent**  **DVT** | 0 | 4  (2.8) | NR | 0* | 0 | 3 | 1254 | 35.1 |
| **Recurrent**  **PE** | 0 | 1 | NR | 0* | 0 | 3 | 5314 | 37.2 |
| **All-cause mortality** | 23  (15.8) | 15  (10.5) | NR | 23 | 0.07 | 11 | 111 | 9.4 |
| **Major Bleeding** | 0 | 2  (1.3) | 0.93  [0.43-2.02] | 0* | 0 | 41° | - | - |
| **CRNMB** | 9  (6.2) | 7  (4.8) |  | 2* | 0.006§ | 11° | - | - |

# **Supplementary Table 7:** Robustness of outcomes from the CASTA DIVA trial. ARR: Absolute Risk reduction; CI: Confidence interval; CRNMB: Clinically relevant non-major bleeding; DVT: Deep vein thrombosis; FI: Fragility index; FQ: Fragility quotient; HR: Hazard ratio; NNH: Number needed to harm; NNT: Number needed to treat; NR: Not reported PE: Pulmonary embolism; RFI: Reverse fragility index; RRR: Relative Risk Reduction; VTE: Venous thromboembolism; *Calculated as RFI. §Calculated as RFQ.

|  | **CASTA DIVA** | | | | | | | |
| --- | --- | --- | --- | --- | --- | --- | --- | --- |
|  | **Rivaroxaban**  **N=74** | **Dalteparin**  **N=84** | **HR**  **[95% CI]** | **FI/**  **FRI*** | **FQ/**  **RFQ§** | **NNT**  **or NNH°** | **RRR (%)** | **ARR (%)** |
| **Recurrent**  **VTE** | 4  (6.4) | 6  (10.1) | 0.75  [0.21-2.65] | 1* | 0.006§ | 59 | 24 | 1.7 |
| **Recurrent**  **DVT** | 0 | 3  (3.5) | NR | 0* | - | 45 | 61 | 2.2 |
| **Recurrent**  **PE** | 1  (1.3) | 2  (2.3) | NR | 0* | - | 100 | 42 | 1 |
| **All-cause mortality** | 19  (25.7) | 20  (23.8) | 1.05  [0.56-1.97] | 5* | 0.03* | 53 | 8 | 1.9 |
| **Major Bleeding** | 1  (1.4) | 3  (3.7) | 0.36  [0.04-3.43] | 0* | - | 45° | - | - |
| **Major Bleeding +**  **CRNMB** | 9  (12.2) | 8  (9.8) | 1.27  [0.49-3.26] | 5* | 0.03* | 37° | - | - |

# **Supplementary Figure 1.** Risk of Bias Assessment of Selected Trials using the Cochrane risk of bias tool for randomized trials (Version 2).

# **Supplementary Figure 2.** Forest plot of outcomes from the included randomized controlled trials. CRNMB: Clinically relevant non-major bleeding; DVT: Deep vein thrombosis; PE: Pulmonary embolism; VTE: Venous thromboembolism.

# **Supplementary Figure 3.** Funnel plot for the main outcomes of the considered randomized controlled trials.
